# Tumor draining lymph nodes in non-small cell lung cancer: *underrecognized role in biomarker discovery and therapeutic innovation*

**DOI:** 10.3389/fimmu.2026.1781682

**Published:** 2026-04-23

**Authors:** Alexander M. Bain, Andrew J. DeMaio, Antonio Velez, Jun-Chieh J. Tsay, Daniel H. Sterman

**Affiliations:** 1 Division of Pulmonary, Critical Care & Sleep Medicine, New York University Grossman School of Medicine, New York, NY, United States; 2 NYU Pulmonary Oncology Research Team (NYU PORT), New York University Grossman School of Medicine, New York, NY, United States; 3 Division of Pulmonary and Critical Care Medicine, Jackson Health System, Miami, FL, United States; 4 Division of Pulmonary and Critical Care Medicine, VA New York Harbor Healthcare System, New York, NY, United States

**Keywords:** cancer immunology, immunotherapy, lymph node metastases, non small cell lung cancer (NSCLC), TDLN, tumor-draining lymph node

## Abstract

Recent advances have furthered our understanding of the role of the tumor draining lymph node (TDLN) in the immune response to thoracic malignancies. This review synthesizes the rapidly expanding evidence that tumor draining lymph nodes (TDLNs) are not passive conduits of metastasis but dynamic immunologic organs that shape anti-tumor immunity in non–small cell lung cancer (NSCLC). Across cytokine, cellular, genomic, transcriptomic, and metabolic domains, the TDLN microenvironment becomes progressively remodeled towards immune suppression. These changes influence tumor growth and early metastasis, and may dictate responsiveness to various treatment modalities. The TDLN is also a practical and clinically relevant site for biomarker discovery and therapeutic innovation as a target of drug delivery and immunomodulation.

## 1 Introduction

Over the last century, the lymph node has been established as an important site for the generation of anti-tumor immune responses and for its role in immunosuppressive microenvironment and metastasis (1–3).

In non-small cell lung cancer (NSCLC), nodal status remains an essential determinant of prognosis and treatment. Compared to the primary tumor, relatively few studies have characterized the microenvironment of the lymph node in NSCLC. In this review, we examine the role of the tumor draining lymph node (TDLN) in NSCLC with a focus on its microenvironment and its potential role as a biomarker and target of therapy.

## 2 Structure and function of the lymph node

Tumor draining lymph nodes include the sentinel lymph node (SLN), the most proximate lymph node draining a tumor, as well as other regional lymph nodes that receive lymphatic flow from a tumor bed. In the context of NSCLC, most authors delineate the TDLN based on established drainage patterns within the lung (4).

**Figure 1:**
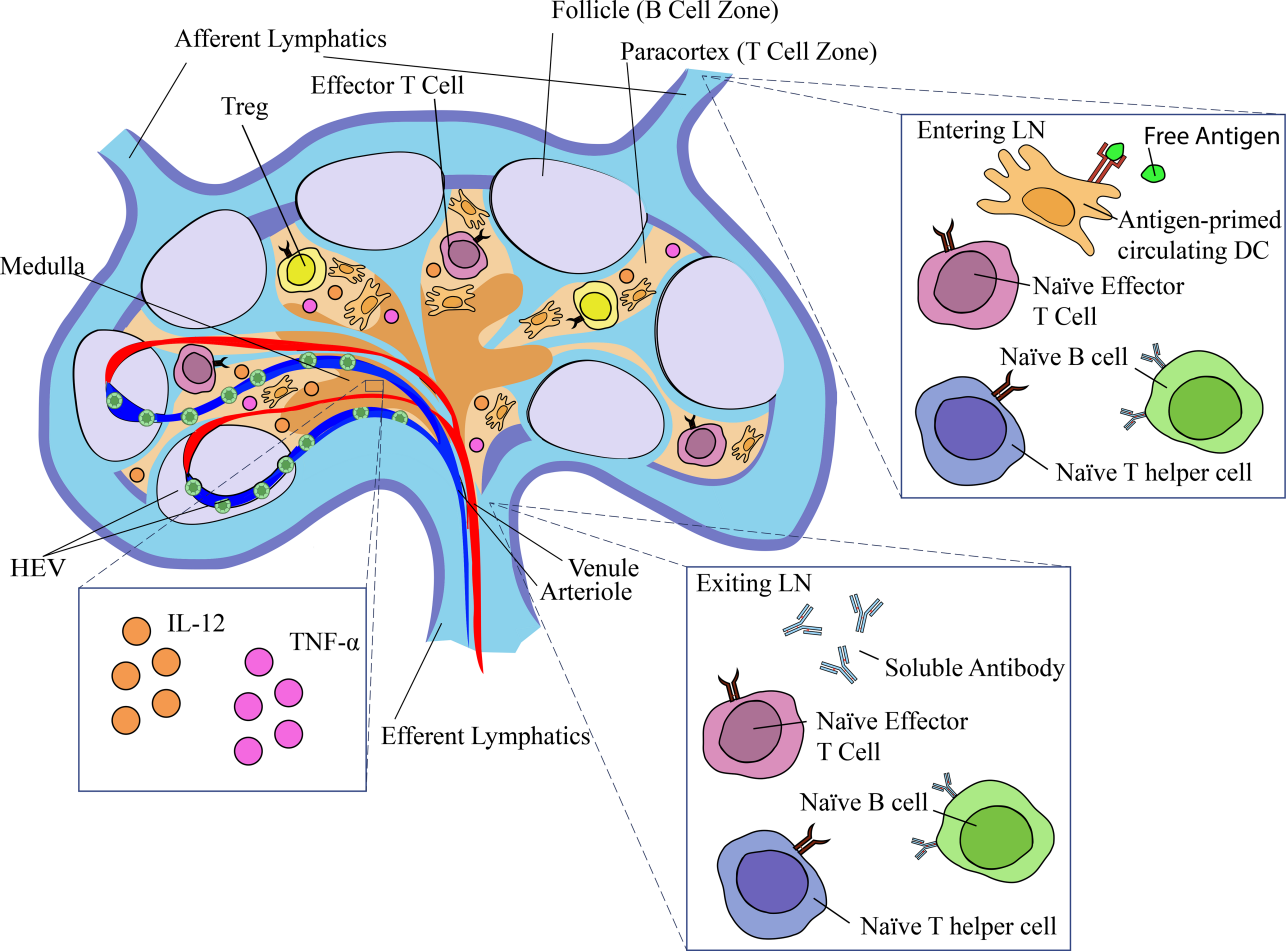
Structure and function of the lymph node in NSCLC and mechanisms of immunosuppression. Afferent lymph enters at the top of the lymph node where it encounters CD169+ macrophages in the subcapsular sinuses. Lymphatic fluid flows periphery to the center of the node where it is sampled by antigen-presenting cells in the cortex, flows through medullary sinuses, and eventually exits the lymph at the hilum. The nodal immune environment is modified by cytokines, exosomes, and blood and lymphatic growth factors.

The cortex is located peripherally and contains lymphoid follicles comprised mostly of B cells. Naïve B cells recruited by chemokines such as CXCL13 migrate to distinct zones, where they form germinal centers and produce antibodies to an almost limitless repertoire of antigens (**Figure 1**) (5). The paracortex, or T-cell zone, is the major site of antigen presentation and formation of cell-mediated immunity. Running through the paracortex are specialized blood vessels called high-endothelial venules (HEV), which express cell adhesion molecules and secrete the chemoattractants CCL19 and CCL21. HEVs allow CCR7+ lymphocytes, including antigen presenting cells (APCs) and T cells, to selectively enter the node from the bloodstream (6). The complex interactions in the T-cell zone that generate the adaptive immune response have been described at length (5, 7, 8).

Tertiary lymphoid structures (TLSs), though not strictly lymph nodes, are also involved in anti-tumor immunity. TLSs form in the tumor and at the invasive margins of several cancer types, and are sites of antigen presentation, T cell priming, and generation of anti-tumor immunity (9). TLS density is associated with improved prognosis in NSCLC (9, 10). TDLNs may influence the maturation of TLS within the primary tumor, and tumor-invaded TDLNs are associated with decreased GC formation within the tumor, decreased B cell infiltration, and diminished memory B cell formation (11).

## 3 The immunologic role of the TDLN

Many key interactions among immune and tumor cells occur in the TDLN, and under ideal conditions, result in the production of immunity against the tumor. Tumor-derived antigens are processed and presented to the naïve immune system in the TDLN, and its environment has a strong influence on whether immune activation or tolerance occurs (12).

Tumor-induced changes in the TDLN environment suppress immune surveillance and enable malignant proliferation. Coordinated remodeling of the TDLN environment by tumor cells leads to a “pre-metastatic niche.” (13–15) This process includes both antigen and non-antigen mediated pathways. Previously described mechanisms include lymphatic vessel remodeling (16), secretion of growth factors, immunosuppressive cytokines and exosomes (3, 17), alterations of immune cell populations (18) and defects in antigen presentation (19, 20). The underlying mechanisms have been reviewed in detail, with most data coming from melanoma and breast cancer (3, 12, 13, 21, 22). Each of these changes can precede development of metastatic disease in the TDLN. Tumor-induced alterations in the TDLN microenvironment are a necessary precondition of nodal metastases via lymphatic flow.

Early studies suggested that lymphocytes isolated from TDLN are more active than lymphocytes in other compartments, with increased IL-2 production and cytotoxicity (23, 24). But when compared to metastatic nodes, TDLN without metastases had a higher ability to produce IL-2 upon stimulation (23). More recent work by Zhang et al. demonstrates an immune-suppressed environment in metastasis-positive TDLNs with reduced dendritic cell (DC) maturation, accumulation of Tregs, reduced effector T cells, and increased anergic CD4 T cells (25). Put together, these studies suggest a dynamic microenvironment with increasing immune suppression with more advanced tumors.

## 4 Early metastasis to the TDLN

When tumor cells disseminate to the lymph node, they may be destroyed, continue their journey into the efferent lymphatic fluid, or remain in the lymph node and form metastases. Assessment of nodal metastasis is among the most important factors in NSCLC staging and prognostication. Advances in endobronchial ultrasound with transbronchial needle aspiration (EBUS TBNA) have enabled minimally invasive and highly accurate mediastinal LN staging (26). As a result, TDLN in thoracic malignancies are notable due to their reliable and routine accessibility using modern techniques.

If malignant involvement is detected on LN histology or cytology, immunohistochemistry (IHC) is performed to further determine histologic subtype and evaluate PD-L1 expression to assess candidacy for immunotherapies. Other molecular methods including next generation sequencing are utilized to evaluate for targetable mutations in NSCLC, including in thoracic small biopsy specimens.

The SLN is the first lymph node to receive lymphatic flow from a tumor, and thus is often an early site of metastasis in many cancer types (27). When a tumor metastasizes to a lymph node, malignant cells typically first occupy the subcapsular sinus, then progressively invade the medullary sinus, medulla, and cortex until the process culminates in complete replacement of the lymph node parenchyma (28). Recent studies highlight the importance of macrophages in the subcapsular sinuses in host defense. These immune cells, often identified by CD169 expression, are the first immune cells to encounter afferent lymph and are involved in immune responses to infections and tumors (29). During the progression of metastasis, frequent changes in the tumor and lymph node environment are noted, driven by many biologically active molecules released from the tumor itself and the surrounding stromal cells (including epithelial and fibroblast growth factors, VEGF, TNF-α, TGF-β, interleukins, chemokines, proteases) (30). Metastatic tumor cells in the TDLN can subsequently invade blood vessels and metastasize to distant organs (31, 32). Many of the mechanisms of this dynamic process remain incompletely understood.

Nodal micrometastases (NMM) have been associated with a worse prognosis in NSCLC.

Several recent studies in NSCLC have associated micrometastases (defined as tumor deposits between 0.2 mm and 2 mm in greatest dimension) with reduced disease-free and/or overall survival (33). Even the presence of isolated tumor cells (defined as single or clusters of malignant cells < 0.2 mm) may also hold negative prognostic value, although this is more controversial (34–36). Compared to TDLNs with micrometastases, lymph nodes with macrometastases demonstrate dysregulation of molecules involved in the epithelial-to-mesenchymal transition (EMT) in patients with NSCLC (37). Other researchers have found increased immune activation and memory in malignant-negative TDLNs in otherwise metastatic patients when compared to patients with malignant-positive TDLNs and patients with no metastases and negative TDLNs (25).

## 5 The lymph node environment in NSCLC

The TDLN microenvironment in NSCLC is shaped by distinct cytokine, cellular, genomic, transcriptomic, and metabolic features that influence immune response and disease progression.

### 5.1 Cytokine involvement in the nodal microenvironment

A number of cytokines have been implicated in the immunosuppressive microenvironment of tumors and TDLN. TGF-β—an immunosuppressive cytokine with diverse roles in cellular proliferation, differentiation, and apoptosis—has been associated with an altered immune tumor environment (38). In a study of patients undergoing resection for NSCLC, TGF-β levels were significantly higher in the TDLN versus the non-draining lymph node (NDLN), although both were 30-fold less than in the tumor itself (39). This gradient across the tumor microenvironment reinforces the tumor as the source of elevated TGF-β. Other studies in lung cancer have corroborated the role of TGF-β in nodal immune suppression. Bugalho et al. evaluated the TDLN of patients with NSCLC during staging using EBUS TBNA and found an increase in the immunosuppressive cytokines TGF-β and IL-10, and a decrease in the inflammatory cytokines IL-12 and TNF-α (40). IL-10 expression from the TDLN correlated with tumor stage, and IL-12 expression from the TDLN correlated inversely with survival.

Saeed et al. identified a novel cytokine profile associated with metastasis of NSCLC to TDLNs using EBUS TBNA (41). Analysis of a panel of 34 cytokines relevant to cancer biology found a significant increase in concentrations of sVEGFR-1, IL-6, VEGF-A, angiopoietin-2, uPA, sHER-2/neu, and PLGF in malignant lymph node samples. Additionally, the vascularity of metastatic lymph nodes on EBUS has been correlated with expression of HIF-1a and VEGF-C (42), while other investigators have found an increase in cytokeratin-19 (CK-19), carcinoembryonic antigen (CEA), and epithelial cell adhesion molecule (EpCAM) in TDLN versus benign lymph nodes (43).

### 5.2 Cellular involvement in the nodal microenvironment

The implications of varying cellular compositions of the TDLN microenvironment has been the subject of multiple studies in NSCLC (see **Table 1**). Compared to benign lymph nodes, TDLN from patients with NSCLC demonstrate a decreased overall percentage of immune cells, decreased relative abundance of lymphocytes, and increased monocytes. Myeloid APCs are increased but with decreased CD80/CD86 expression, suggesting that the APCs in the TDLN have a decreased costimulatory capacity (40).

**Table 1:** Recent studies evaluating the lymph node environment in NSCLC.

Authors/Year	Significant findings in TDLN	Comparator	TBNA specimens
Ito et al. / 2006 (39)	Increased DC apoptosis, TGF-β Decreased DC	Non-SLN*	No
Tabarkiewicz et al. / 2008 (46)	Increased lymphoid/plasmacytoid (CD303^+^) DC to myeloid (CD1c^+^) DC ratio	Primary tumor, PBMC*	No
Schneider et al., 2011 (49)	Increased Treg cells in NMLN of adenocarcinoma samples	Primary tumor, TDLN	No
Nakajima et al. / 2012 (42)	Increased HIF-1α, VEGF-A mRNA	Benign LN*	Yes
Bugalho et al. / 2013 (43)	Increased monocytes and epithelial cells, CK-19, CEA, EPCAM mRNA Decreased lymphocytes	Benign LN	Yes
Bugalho et al. / 2016 (40)	Increased monocytes (CD45^+^/moderate SSC), TGF-ß, IL-10 Decreased immune cells (CD45+), lymphocytes (CD45+/low SSC), TNF-α, IL-12; CD80/86 by mAPC (CD45*/MHC-IF/CD19)	Benign LN	Yes
Aramaki et al. / 2016 (37)	Increased SMA^+^ fibroblasts, CD34+ microvessels, CD204+ macrophages Decreased E-cadherin	Micrometastatic LN* (< 2 mm)	No
Pogoda et al. / 2016 (51)	Increased monocytes/macrophages (vs PBMC), increased IL-10 production by monocytes/macrophages (vs MDSC)	Primary tumor, TDLN, PBMC*	No
Domagala-Kulawik et al. / 2017 (50)	MPLN with reduced CD8+ and higher FOXP3/CD8	MNLNs, NMLNs	No
Saeed et al. / 2017 (41)	Increased sVEGFR-1, IL-6, VEGF-A, angiopoietin-2, uPA, sHER-2/neu, and PLGF	Benign LN	Yes
van de Ven et al. / 2017 (44)	Increased PD-1^+^ cells, PD-1 expression on CD4^+^ and CD8^+^ T cells	PBMC, NDLN*	Yes
Sappington et al. / 2018 (72)	Increased kynurenine to tryptophan ratio Decreased reduced and oxidized glutathione levels	Benign LN	Yes
Murthy et al. / 2019 (45)	Increased T regulatory cells Decreased CD4^+^ T cells	PBMC, NDLN*	Yes
Stankovic et al. / 2019 (52)	Increased CD45^+^ leukocytes, CD19^+^ B cells, CD27^+^CD38^+^/- B cells, naïve B cells (vs tumor and distal lung). Decreased macrophages/monocytes (vs tumor and distal lung)	Primary tumor, distal lung, PBMC*	No
Yang et al. / 2022 (75)	Decreased pCR-P/MPR-P, decreased RFS and OS Decreased CD8+ T cell infiltration, increased FOXp3+ Treg cells	MNLNs, NMLNs	No
He et al. / 2023 (11)	Less GC formation in TLS of MPLNs Reduced B cell infiltration, memory B cell formation	Primary tumor (TLS), MNLNs	No
Khalifa et al., 2023 (78)	Increased memory T cell subsets Decreased B cell-receptor signaling	CRT+ TDLN, CRT- TDLNs	No
Borm et al. / 2023 (48)	Decreased PD-1hi CD8+ T cells and PD-1hi Tregs post ICI	PBMCs	Yes
Engin et al. / 2023 (80)	Decreased PD-1, CTLA-4 expression Improved granzyme A expression	MNLNs, NMLNs	No
Cikman et al. / 2023 (81)	Improved ratio of effector to exhausted NK cells Recovery of cytotoxic activity of NK cell (increased LAMP-1, Granzyme A levels)	PBMCs	No
Zhang et al. / 2024 (25)	Greater TCR clonal diversity in MNLN Increased CD8+ T-cell activation and memory	Primary tumor, MPLN, NMLN	No
Ulas et al. / 2025 (79)	Increased CD8 and Treg cells, increased type I immune responses in immunotherapy group. Immune response persisted even in TDLN with radiation-induced fibrosis	CRT +/- immunotherapy TDLNs	No

*denotes comparison within the same patient; SLN, sentinel lymph node; PBMC, peripheral blood mononuclear cells; DC, dendritic cell; MDSC, myeloid derived suppressor cell; TNF-α, tumor necrosis factor α; TGF-β, transforming growth factor β; IL-6, interleukin-6; IL-10, interleukin-10; IL-12, interleukin-12; sVEGFR-1, soluble vascular endothelial growth factor receptor 1; VEGF-A, vascular endothelial growth factor A; PLGF, placental growth factor; uPA, urokinase-type plasminogen activator; HIF-1α, hypoxia inducible factor 1α; SMA, smooth muscle actin; CK-19, cytokeratin 19; CEA, carcinoembryonic antigen; EPCAM, epithelial cell adhesion molecule; PD-1, programmed cell death protein 1; SSC, side scatter; MNLN, metastasis-negative lymph nodes from LN-metastatic patients; MPLNs, metastasis-positive lymph nodes; NMLNs, TDLNS from non-LN-metastatic patients; TLS, tertiary lymphoid structure; CRT, chemoradiotherapy; ICI, immune checkpoint inhibitor; pCR-P, pathological complete response in primary tumor bed; MPR-P, major pathological response in tumor bed; RFS, recurrence-free survival; OS, overall survival.

van de Ven et al. demonstrated increased frequency of PD-1+ effector and regulatory T cells (Tregs) in the TDLN compared to the peripheral blood. This finding raises the possibility of using PD-1 expression levels of immune cells as a biomarker of immune checkpoint blockade responsiveness (44). Murthy et al. performed flow cytometry to quantify immune cell populations in patients with suspected NSCLC undergoing bronchoscopic diagnosis and staging and found that the TDLN had decreased CD4+ effector T cells and increased Tregs. Further, patients with PD-L1 ≥ 50% measured in the primary tumor had greater regional CD4+ T cell depletion than patients with PD-L1 < 50% (45).

Dendritic cell counts within TDLN of patients with NSCLC are reduced when compared to NDLN (39). Tabarkiewicz et al. compared DC subsets in peripheral blood, TDLN and primary tumor in 50 patients undergoing resection for NSCLC, and showed an increased lymphoid/plasmacytoid (CD303+) DC to myeloid (CD1c+) DC ratio (suggesting increased tumor escape) in the TDLN when compared to the tumor and peripheral blood (46). Tumor-infiltrating plasmacytoid DC have previously been shown to be associated with a poor prognosis through induction of immune tolerance (47).

In a unique human study in NSCLC comparing flow-cytometric T cell profiles in patients treated with ICIs, Borm et al. found increased Treg frequencies in TDLNs of progressors compared to responders (48). Larger studies are necessary to further investigate the role of TDLNs as targets and prognostic indicators to ICI therapy.

There are also potential differences in the TDLN cellular composition based on histologic subtype of NSCLC. In one cohort, TDLNs exhibited marked FOXP3^+^ Treg accumulation that was more pronounced in adenocarcinoma than in squamous cell carcinoma, accompanied by reductions in natural killer cells, consistent with a more strongly Treg-skewed immunosuppressive program in adenocarcinoma (49). Similarly, an analysis of resected mediastinal lymph nodes showed that adenocarcinoma nodes with metastasis had reduced CD8^+^ proportions and a higher FOXP3/CD8 ratio compared with adenocarcinoma nodes without metastasis, whereas analogous differences were not observed in squamous cell carcinoma nodes (50).

Other studies have described the role of additional cell populations in the TDLN in patients with NSCLC and are listed in **Table 1** (37, 51, 52).

### 5.3 Genomic alteration relevance in the nodal microenvironment

Lung cancers exhibit a high degree of chromosomal instability and evolve into clonal and subclonal populations with different genomic features in regions of the same tumor (53). Lung cancers also exhibit inter-tumoral heterogeneity between the primary tumor and metastatic lesions, such as those found in lymph nodes. Numerous studies have shown differences in driver mutations and PD-L1 expression between primary lung tumor and metastatic lymph nodes (54–56). These differences may be responsible for differential response to treatment seen with targeted and immune-based therapies and suggest limitations to current biomarkers such as PD-L1 IHC. Zhang et al. have also examined differences in T cell clonal diversity in TDLNs compared to tumor or blood samples and found greater T cell clonal diversity and greater monoclonal expansion in the tumor site (25). While there is no direct study of the differences in TDLN environment stemming from heterogenous driver mutations, the lack of an overall survival benefit of checkpoint inhibitors in *EGFR* mutant tumors raises the prospect that tumor genotype could more broadly influence TDLN conditioning (57).

Further characterization of intra- and inter-tumoral heterogeneity via the TDLNs may eventually help with biomarker development and selection of combination therapies.

### 5.4 Transcriptomic alteration within the nodal microenvironment

Significant advances in specimen retrieval and molecular assays have enriched our understanding of the transcriptomic environment of the TDLN. Early studies used molecular methods to detect mRNA expressed preferentially (or exclusively) in malignant cells to evaluate for nodal micrometastasis in NSCLC (34, 58).

More than a decade ago, Nakajima et al. established the feasibility of RNA expression analysis of samples obtained by EBUS TBNA with microarrays (59). Subsequently, Um et al. investigated the difference in genomic and transcriptomic differences between metastatic LNs and primary tumor using bulk RNA sequencing and found that differential gene expression between primary tumor and metastatic LNs increased with distance from the primary site. This finding demonstrates the need for samples of multiple tumor and metastatic regions to appreciate transcriptomic heterogeneity (60).

Kim et al. performed single-cell RNA (scRNA) sequencing in treatment-naïve metastatic NSCLC specimens and showed that metastatic lymph nodes (including TDLNs) exhibit marked immune remodeling, with enrichment of myeloid populations (including monocyte-derived macrophages and plasmacytoid dendritic cells) and expansion of suppressive cell states (including Tregs and myofibroblast-like stromal cells). Their analyses suggest that within metastatic LNs, macrophages may deliver growth- and immune-suppressing signals (e.g., via TNF/TGFβ and EGFR-activating ligands) to tumor cells—highlighting pathways that could be therapeutically targeted (61).

Most recently, Mu et al. performed scRNA sequencing of malignant epithelial cells within lung adenocarcinoma metastatic lymph nodes, identifying distinct malignant epithelial phenotypic clusters. Using marker genes from prognostically adverse malignant epithelial subpopulations identified in this single-cell framework, they derived an epithelial-associated prognostic risk score and validated its association with overall survival in external cohorts. In the external cohort high-risk tumors, there was increased recruitment/infiltration of granulocytic populations (including neutrophils/eosinophils) and upregulation of proliferative/cell-cycle and metabolic programs (E2F targets, glycolytic metabolism, G2/M checkpoint) (62).

Further studies are warranted to better validate and classify gene expression pathways that are dysregulated in TDLN with metastasis, which may provide novel targets of therapy. Gene expression of TDLN cells may also serve as a readily accessible biomarker to predict response to immunotherapy, as it has in the primary tumor (63).

### 5.5 Metabolic alteration within the nodal microenvironment

Metabolic profiling is another potential target for characterizing and stimulating the anti-tumor immune response of the TDLN. Recent pre-clinical metabolomic studies provide insight into future therapeutic targets for TDLN based interventions. Tryptophan metabolism plays important roles in the regulation of immune suppression. Indoleamine-2,3-dioxygenase 1 (IDO1), an enzyme that regulates the first step in tryptophan catabolism via the kynurenine pathway, is overexpressed in several tumors and other cells in the tumor microenvironment (64). IDO1 induces immunosuppression through several mechanisms, including reprogramming of naïve T cells to Tregs and reducing T cell effector function (65). A pre-clinical model has identified IDO upregulation in antigen presenting cells in the TDLN, highlighting the role of the lymph node in generation of anti-tumor immunity (66). IDO+ Plasmacytoid DCs in mouse TDLNs that are IDO+ activate Tregs and upregulate PD-L1, leading to a suppressive TDLN microenvironment (67).

Glutamine metabolism is another potential therapeutic target of anti-cancer immunotherapy in TDLNs. Glutamine is an important energy source for tumor cells, and metabolic reprogramming of glutamine signaling contributes to immune evasion in the tumor microenvironment via limiting immune cell glutamine utilization and regulating tumor PD-L1 expression (68, 69). However, we are not aware of studies to date that explore the role of glutamine in the TDLN specifically.

There is also emerging evidence for the role of asparagine and asparagine synthetase (ASNS) in immune activation in lung cancer. In a murine model of lung cancer TDLNs, Zhang et al. found that elevated asparagine synthetase (responsible for the conversion of aspartate and glutamine to asparagine) was associated with increased immunogenicity via expression of major histocompatibility complex through α-aminobutyric acid auto-secretion in lung cancer cells. In a human cohort of 25 patients, the same investigators found that high ASNS expression in LN metastases correlated with improved efficacy of neoadjuvant immunotherapy (70).

Arginase overexpression leads to depletion of intratumoral L-arginine, thus suppressing T-cell proliferation while fueling tumor growth through polyamine biosynthesis. In preclinical models, treatment with a dual arginase inhibitor OATD-02 led to an immunostimulatory state via increased intratumoral L-arginine, increased immune cell infiltration, and improved response to immune checkpoint blockade (71).

In a human study examining the metabolic phenotype of lymph node aspirates in patients with NSCLC versus non-malignant conditions, no single metabolomic feature was able to differentiate malignant status or histologic type, but differences were seen in metabolites including kynurenine and oxidized glutathione (72). While no single biomarker of immune tone or treatment responsiveness has emerged across the spectrum of molecular studies, TDLN samples obtained through EBUS TBNA may allow future clinicians to obtain a more complete profile of the microenvironment to guide targeted therapies.

## 6 TDLN as an immunologic target

Due to their accessibility and integral role in the immune response to NSCLC, TDLNs can serve as potential biomarkers of treatment response and targets for immune directed therapies.

Prior research has demonstrated the importance of intact TDLNs to the response to PD-1 blockade (73). In a proteomic analysis of TDLNs in mice treated with anti-PD-1 therapy, Ho et al. found differences in cytokine profiles and cellular remodeling, with enhanced remodeling of B and T cell compartments toward memory phenotypes (74). Yang et al. investigated the role of the TDLN in the response to neoadjuvant ICIs in patients with NSCLC. Neoadjuvant ICI reduced FDG avidity within the tumor bed but not in their respective associated TDLNs. The presence of tumor-invaded TDLNs was associated with poor pathological responses and predictive of rapid post-treatment relapse. Spatial profiling showed exclusion of T cell infiltrates within the metastases of tumor-invaded TDLNs (75).

Some research suggests that radiotherapy may stimulate a distant antitumor immune response via the abscopal effect. This possibility has led to interest in the role of radiotherapy, with and without immunotherapy, directed at the TDLN. In a pre-clinical model, irradiation of the TDLN has been shown to attenuate the adaptive immune effects of tumor radiotherapy via altered chemokine expression and CD8^+^ T-cell trafficking (15, 76). The effect of radiotherapy on TDLN anti-tumor immunity is likely sensitive to timing; recent work by Telarovic et al. suggests that delayed adjuvant irradiation of TDLN may improve the efficacy of radio-immunotherapy (77). In patients who underwent neoadjuvant lymph node sparing chemoradiation (CRT^+^), Khalifa et al. performed bulk RNA sequencing of TDLNs and demonstrated increased expression of gene pathways associated with antitumor immune response, inflammatory response, hypoxia, angiogenesis, extracellular matrix remodeling, and EMT when compared to CRT^-^ controls (78). Most recently, resected TDLNs from patients in the INCREASE trial who underwent neoadjuvant immunotherapy plus chemoradiotherapy demonstrated enhanced type I immune responses as well as increased CD8 and regulatory T cells in irradiated TDLN when compared with non-irradiated controls (79).

These findings suggest that the TDLN is an important site for generating an anti-tumor T cell response, and while irradiation of the primary tumor may induce an antitumor immune response at the TDLN level, direct TDLN irradiation has a detrimental effect on anti-tumor immune activation.

Recent work has also examined how surgical removal of TDLNs may impact systemic immune tone. Engin et al. explored cytotoxic T-lymphocyte (CTL) expression patterns in mediastinal lymph nodes (MLN) and reported changes in CTLs from peripheral blood after video-assisted mediastinoscopic lymphadenectomy (VAMLA). VAMLA was associated with a reduction in PD-1 and CTLA-4 expression in peripheral CTLs, as well as an improvement in Granzyme A expression. These findings may help explain at a cellular level a rationale for improved survival after LN dissection in NSCLC (80). In another cohort of patients who underwent VAMLA, Cikman et al. found increased effector NK cell expression, decreased exhausted NK cell subset, and increase in cytotoxic activity (via LAMP-1 and Granzyme A) in PBMCs (81).

Cryoablation of primary lung tumors has long been of interest due to its potential for induction of antitumor immune responses and synergy with immunotherapy (82). In addition to direct cell injury and localized vascular thrombosis, cryoablation induced necrosis leads to the release of tumor antigen and pro-inflammatory cytokines including IFN-γ and TNF- α. More recently, bronchoscopic cryoimmunotherapy (BCI) targeted at peripheral lung tumors has emerged as a promising area of investigation; expansion to intra-nodal BCI represents a logical next step in therapeutic trials (83).

Specific immune cells within the TDLN may be targeted using drug delivery systems for effective therapies with fewer off-target effects. For example, antigen presenting cells may be targeted through the injection of nanoparticle-bound antigens into the dermal or subcutaneous tissue near the lymph node of interest. Depending on particle size, they may be transferred through lymphatic vessels to the lymph node where they will be taken up by antigen-processing cells. Larger particles may instead be processed by peripheral DCs and subsequently transported to the lymph node. Alternatively, T cells in the paracortex can be targeted by conjugating an antibody to the homing molecule CD62L (selectin) to promote its uptake through high endothelial venules (84). A therapeutic agent may also be injected directly into the lymph node via EBUS guidance where it may accumulate at higher concentrations than through systemic delivery. Potential targets of intranodal therapy include intranodal vaccination with lipid nanoparticle (LNP)-mRNA constructs (85, 86) and intranodal delivery of tumor-antigen pulsed dendritic cells (87).

Other researchers have used cells from TDLN for adoptive immunotherapy in NSCLC (88). TDLN without nodal metastasis are a potent source of tumor-specific DCs and killer cell precursors, which are activated in the presence of IL-2. A small single-center randomized controlled trial (performed prior to the current era of targeted and immunotherapies) showed improved survival for patients receiving adjuvant adoptive immunotherapy (89). This trial highlights the potential of adoptive cell therapy, which warrants further evaluation.

## 7 Discussion

Recent advances have furthered our understanding of the role of the TDLN in the immune response to thoracic malignancies.

Our current knowledge underscores the immunosuppressive milieu within the TDLN that drives tumor progression. In terms of mechanistic studies, unraveling immune dysregulation will require advanced approaches (B/T cell receptor sequencing, single-cell RNA sequencing, high-parameter flow cytometry, and multi-omic analyses) to define how immune cell types in the TDLN interact and signal across space and time. Further understanding the environment and function of the TDLN may provide opportunities for novel therapies targeted at the TDLN.

Looking into the future of clinical studies, we must build on early attempts to develop individualized risk stratification tools based on immune profile phenotyping of the TDLN. We must also advance the compelling frontier of therapeutic interventions targeting the TDLN in NSCLC management. The relative ease of access of the TDLN in patients with lung cancer has enabled TDLN to be included as sites for potential delivery of experimental therapeutic agents in currently ongoing trials including such as viral immunotherapy (NCT04495153) (90), multi-armed oncolytic viruses (NCT06311578) (91), and hafnium radiosensitizers (NCT04505267) (92). Whether through precision drug delivery systems or adoptive immunotherapy strategies harnessing the TDLN’s immunomodulatory potential, innovative approaches hold the potential to reshape treatment paradigms and improve patient outcomes. By continuing to interrogate the complexities of the TDLN, we pave the way for transformative advancements in NSCLC therapeutics and personalized medicine.
